# Supplementary material for: Wearable technology to inform the prediction and diagnosis of cardiorespiratory events: a scoping review
Source: PeerJ. 2021 Dec 22;9:e12598. doi: 10.7717/peerj.12598 (PMC8710054; doi:10.7717/peerj.12598)
Supplement: Supplemental Information 1 [file peerj-09-12598-s001.docx]

**Supplementary File 1:** Search strategy used for each electronic database searched.

**CINAHL**

| Search | Search String |
| --- | --- |
| #1 | (TI "smart textile*" OR AB "smart textile*" OR TI e-textile OR AB e-textile OR TI e-textiles OR AB e-textiles OR TI "electronic textil*" OR AB "electronic textil*" OR TI "wearable device" OR AB "wearable device" OR TI "wearable technology" OR AB "wearable technology" OR TI "wearable sensor" OR AB "wearable sensor" OR (MH "Wearable Electronic Devices+") NOT Hearing) |
| #2 | ((MH "Diagnosis+") OR TI diagnosis OR AB diagnosis OR TI detect* OR AB detect* OR TI "decision support system" OR AB "decision support system" OR decision-making) |
| #2 | (TI cardiac OR AB cardiac OR TI respiratory OR AB respiratory OR TI respirat* OR AB respirat* OR TI cardiorespiratory OR AB cardiorespiratory OR TI febrile OR AB febrile OR TI Physiology OR AB Physiology OR (MH "Monitoring, Physiologic+")) |
| #4 | #1 AND #2 AND #3 |

**EMBASE**

| Search | Search String |
| --- | --- |
| #1 | ('smart textile*':ti,ab OR 'e textile':ti,ab OR 'e textiles':ti,ab OR 'electronic textil*':ti,ab OR 'wearable device':ti,ab OR 'wearable technology':ti,ab OR ((wearable NEAR/2 device):ti,ab) OR 'wearable sensor':ti,ab OR 'wearable computer'/exp OR 'wearable computer') NOT ('hearing'/exp OR hearing) |
| #2 | (diagnosis:ti,ab OR detect*:ti,ab OR 'decision support system':ti,ab OR 'decision making':ti,ab OR algorithm*:ti,ab OR clinical:ti,ab) |
| #3 | (cardiac:ti,ab OR respiratory:ti,ab OR respirat*:ti,ab OR cardiorespiratory:ti,ab OR febrile:ti,ab OR patients:ti,ab OR physiology:ti,ab OR telemetry:ti,ab) |
| #4 | #1 AND #2 AND #3 |

**Ovid MEDLINE**

| Search | Search String |
| --- | --- |
| #1 | (smart textile*. ti,ab. OR e-textile.ti,ab. OR e-textiles.ti,ab. OR electronic textil*.ti,ab. OR wearable device.ti,ab. OR wearable technology.ti,ab. OR (wearable.ti,ab. ADJ2 electronic.ti,ab. ADJ2 device.ti,ab.) OR wearable sensor.ti,ab. OR exp Wearable Electronic Devices/ NOT Hearing) |
| #2 | (exp Diagnosis/ OR diagnosis.ti,ab. OR detect*.ti,ab. OR decision support system.ti,ab. OR decision-making OR algorithm*.ti,ab. OR clinical.ti,ab.) |
| #3 | (cardiac.ti,ab. OR respiratory.ti,ab. OR respirat*.ti,ab. OR cardiorespiratory.ti,ab. OR febrile.ti,ab. OR patients.ti,ab. OR Physiology.ti,ab. OR exp Monitoring, Physiologic/) |
| #4 | #1 AND #2 AND #3 |

**PUBMED**

| Search | Search String |
| --- | --- |
| #1 | (smart textile*[tiab] OR e-textile[tiab] OR e-textiles[tiab] OR electronic textil*[tiab] OR “wearable device”[tiab] OR “wearable technology”[tiab] OR (wearable[tiab] ADJ2 device[tiab]) OR “wearable sensor”[tiab] OR "Wearable Electronic Devices"[Mesh] NOT Hearing) |
| #2 | (Diagnosis [Mesh] OR diagnosis[tiab] OR detect*[tiab] OR “decision support system”[tiab] OR decision-making OR algorithm*[tiab] OR clinical[tiab]) |
| #3 | (cardiac[tiab] OR respiratory[tiab] OR respirat*[tiab] OR cardiorespiratory[tiab] OR febrile[tiab] OR patients[tiab] OR Physiology[tiab] OR "Monitoring, Physiologic"[Mesh]) |
| #4 | #1 AND #2 AND #3 |

**SCOPUS**

| Search | Search String |
| --- | --- |
| #1 | (TITLE-ABS ("smart textile")) OR (TITLE-ABS (e-textile)) OR (TITLE-ABS (e-textiles)) OR (TITLE-ABS (electronic AND textil*)) OR (TITLE-ABS ("wearable device")) OR (TITLE-ABS ("wearable technology")) OR (TITLE-ABS (wearable W/2 device)) OR (TITLE-ABS ("wearable sensor")) OR (INDEXTERMS ("wearable electronic devices") AND NOT hearing) |
| #2 | (INDEXTERMS (diagnosis)) OR (TITLE-ABS (diagnosis)) OR (TITLE-ABS (detect)) OR (TITLE-ABS ("decision support system")) OR (TITLE-ABS ("decision-making")) OR (TITLE-ABS ("algorithm")) OR (TITLE-ABS ("clinical")) |
| #3 | (TITLE-ABS ("cardiac")) OR (TITLE-ABS ("respiratory")) OR (TITLE-ABS ("respirat*")) OR (TITLE-ABS ("cardiorespiratory")) OR (TITLE-ABS ("febrile")) OR (TITLE-ABS ("patients")) OR (TITLE-ABS ("physiology")) OR (INDEXTERMS ("Monitoring, Physiologic")) |
| #4 | #1 AND #2 AND #3 |

**SPORTDiscus**

| Search | Search String |
| --- | --- |
| #1 | (TI "smart textile*" OR AB "smart textile*" OR TI e-textile OR AB e-textile OR TI e-textiles OR AB e-textiles OR TI "electronic textil*" OR AB "electronic textil*" OR TI "wearable device" OR AB "wearable device" OR TI "wearable technology" OR AB "wearable technology" OR TI "wearable sensor" OR AB "wearable sensor" OR (MH "Wearable Electronic Devices+") NOT Hearing) |
| #2 | ((MH "Diagnosis+") OR TI diagnosis OR AB diagnosis OR TI detect* OR AB detect* OR TI "decision support system" OR AB "decision support system" OR decision-making) |
| #2 | (TI cardiac OR AB cardiac OR TI respiratory OR AB respiratory OR TI respirat* OR AB respirat* OR TI cardiorespiratory OR AB cardiorespiratory OR TI febrile OR AB febrile OR TI Physiology OR AB Physiology OR (MH "Monitoring, Physiologic+")) |
| #4 | #1 AND #2 AND #3 |

| **Database** | **Search Date** | **Results** |
| --- | --- | --- |
| CINAHL | May 6^th^, 2020 | 133 |
| EMBASE | May 6^th^, 2020 | 1701 |
| Ovid MEDLINE | May 6^th^, 2020 | 1527 |
| Pubmed | May 6^th^, 2020 | 1592 |
| Scopus | May 6^th^, 2020 | 2334 |
| SportDisucs | May 6^th^, 2020 | 2 |
