# Supplementary material for: Wearable technology to inform the prediction and diagnosis of cardiorespiratory events: a scoping review
Source: PeerJ. 2021 Dec 22;9:e12598. doi: 10.7717/peerj.12598 (PMC8710054; doi:10.7717/peerj.12598)
Supplement: Supplemental Information 2 [file peerj-09-12598-s002.docx]

**Supplementary File 2:** Standardized study selection questionnaire.

| **Questions based on eligibility criteria.** | **Yes** | **No** |
| --- | --- | --- |
| Does the study present or use a wrist wearable or smart textile? |  |  |
| Does the presented/used wrist wearable or smart textile monitor physiological parameters? |  |  |
| Does the presented/used wrist wearable or smart textile have integrated sensors? |  |  |
| Do all physiological sensors required for the wrist wearable or smart textile form an integrated unit? |  |  |
| Does the study focus on the use of a wrist wearable or smart textile to predict or diagnose a cardiac or respiratory event? |  |  |
| Does the study meet inclusion criteria? |  |  |
