# Supplementary material for: Wearable technology to inform the prediction and diagnosis of cardiorespiratory events: a scoping review
Source: PeerJ. 2021 Dec 22;9:e12598. doi: 10.7717/peerj.12598 (PMC8710054; doi:10.7717/peerj.12598)
Supplement: Supplemental Information 3 [file peerj-09-12598-s003.docx]

**Supplementary File 3**: Data charting form.

| **Study Characteristics** | |
| --- | --- |
| Author(s) |  |
| Year of Publication |  |
| Title |  |
| Country of Origin |  |
| Study Type |  |
| **Sample** | |
| Study Population |  |
| Sample Size |  |
| **Participants** | |
| Age Range |  |
| Gender – Male / Female |  |
| **Study Details** | |
| Study Aim |  |
| Wearable Technology Used |  |
| Brand |  |
| Physiological Measures Collected |  |
| Technological Method of Biometric Measurement |  |
| Length of Monitoring |  |
| Monitoring Conditions |  |
| Clinical Insight(s) Produced |  |
| Sensitivity and Specificity (if applicable) |  |
| Comparator (If applicable) |  |

Adapted from: Aromataris E, Munn Z (Editors). JBI Manual for Evidence Synthesis. JBI, 2020. Available from [https://synthesismanual.jbi.global](https://synthesismanual.jbi.global/).  <https://doi.org/10.46658/JBIMES-20-01>
